# Efficacy and safety of remimazolam besylate and ciprofol in painless gastrointestinal endoscopy in the elderly

**DOI:** 10.1590/acb399324

**Published:** 2024-12-16

**Authors:** Ziwei Zhu

**Affiliations:** 1 The Fourth Affiliated Hospital of Soochow University – Department of Anesthesiology – Suzhou, Jiangsu – China.

**Keywords:** Endoscopy, Gastrointestinal, Hemodynamics, Cognition, Anesthetics

## Abstract

**Purpose:**

This study aimed to ascertain the efficacy and safety of remimazolam besylate and ciprofol in elderly patients undergoing painless gastrointestinal endoscopy.

**Methods:**

Patients in the control group (n = 31) were anesthetized with ciprofol, and those in the observation group (n = 31) were anesthetized with remimazolam besylate. The anesthetic effect, analgesic effect, hemodynamics, cognitive function, and adverse reactions between the two groups were compared.

**Results:**

The observation group had shorter anesthesia onset time, recovery time, as well as anesthesia recovery room stay time and a higher effective rate of analgesia. At T_1_, T_2_, and T_3_, the mean arterial pressure and heart rate of the observation group were higher versus the control group; at T_1_ and T_2_, SpO_2_ in the observation group was higher versus the control group. The simple mental state scale score of the observation group was higher than that of the control group (all *p* 0.05). The total incidence of adverse reactions exhibited no difference between the two groups (*p*> 0.05).

**Conclusion:**

Versus ciprofol, remimazolam besylate has better anesthetic and analgesic effects, more stable hemodynamics, less impact on patients’ cognitive function, and good safety in elderly patients undergoing painless gastrointestinal endoscopy.

## Introduction

Elderly patients are representatives of a special group of patients with a higher incidence of co-morbid disorders and more susceptibility to endoscopic interventions^1^. Dramatic elevations in life expectancy result in increasing utilization of gastrointestinal endoscopy in elderly patients^2^. Advanced age is one of the significant risk factors for adverse reactions in the process of procedural sedation^3^. However, there are limited studies examining the risks, benefits, and utilization of common endoscopic procedures in the elderly group^4^. Therefore, our study focused on probing the safety and efficacy of remimazolam besylate and ciprofol in the elderly who undergo painless gastrointestinal endoscopy (PGE).

Propofol use is involved in risks such as hypo-tension, respiratory depression and pain at the injection site, and remimazolam and ciprofol attract the interest of researchers who are looking for alternatives to propofol^5^. Remimazolam and ciprofol are also new drugs tried to upgrade total intravenous anesthesia practice^6^. Remimazolam, a novel ultra-short-acting benzodiazepine, has been approved to be employed in procedural sedation, and this drug is also beneficial for both consistent sedation and quick recovery in PGE^7^. Remimazolam is also reported to have a minimal accumulative impact and reduced cardio-respiratory depression^8^. Remimazolam may serve as a viable alternative in elderly patients who undergo endoscopic procedures since it is linked to lower rates of hypo-tension in elderly patients undergoing upper gastrointestinal endoscopy under deep anesthesia or sedation versus propofol^9^. Moreover, remimazolam possesses not only sedative, amnestic, and anxiolytic properties, but also favorable hemodynamic stability^10^. Remimazolam tosilate, implemented in procedural sedation and general anesthesia, can be a safe and effective option for gastrointestinal endoscopy sedation in elderly patients, along with a lower incidence of sedation-relevant adverse reactions^11^. Remimazolam tosilate is feasible and well tolerated for patients with superior safety profile than propofol^12^.

Gastrointestinal endoscopy can be a difficult experience and leave an unpleasant impression on patients. Remimazolam tosylate has mild inhibitory effects on the respiratory system and the circulatory system^13^. Ciprofol refers to an agonist of the γ-aminobutyric acid type A receptor^14^. Versus propofol, ciprofol possesses fewer effects on hemodynamics and is more suitable for painless gastroscopy^15^. Currently, ciprofol is employed in anesthesia induction and PGE, and ciprofol at an appropriate dose is more advantageous for PGE than propofol in hemodynamics along with respiratory stability, with diminished nausea and vomiting, as well as pain at the injection site^16^. Furthermore, ciprofol has pharmacodynamic characteristics of rapid onset and recovery rate in pre-clinical experiments^17^. As a novel intravenous anesthetic agent, ciprofol shows respiratory and hemodynamic stability and provides better sedative effects with raised recovery quality and satisfaction^18^. However, the effects of remimazolam besylate and ciprofol in PGE in the elderly are rarely discussed.

Therefore, this paper was aimed at unveiling the efficacy and safety of remimazolam besylate and ciprofol in elderly patients undergoing PGE.

## Methods

### Ethics statement

The study was under the approval of the Ethic Committee of the Fourth Affiliated Hospital of Soochow University (approval number: 20221215), and the patients and their families offered their written informed consent.

### Study subjects

Sixty-two elderly patients who underwent PGE in the Fourth Affiliated Hospital of Soochow University from March 2023 to June 2023 were recruited as the study subjects, and the subjects were separated into the control and observation groups by random number table method (n = 31 cases). Patients in the control group were anesthetized with ciprofol, and those in the observation group were anesthetized with remazolam benzenesulfonate. Inclusion criteria:

- Patients whose age ≥ 60 years old;
- Those with American Society of Anesthesiologists (ASA) classification I-II;
- Those with complete medical records;
- Those with informed consent for the study.
- Exclusion criteria were:
- Those with a previous history of adverse anesthesia;
- Those combined with severe cardiac, renal, and other organ failure or combined with multiple light failures;
- Those combined with psychiatric disorders or cognitive deficits who could not communicate normally;
- Those with allergies or contraindications to the anesthetic drugs in this study.

### Methodology

Prior to the PGE operation, the patients were fasted for at least 6 hours and deprived of water for at least 2 hours. After the patients entered the examination room, a venous channel was established, and the patients’ routine vital signs were monitored during anesthesia and examination. The patients were supine for oxygen inhalation for 3 minutes (5 L/min), and anesthesia induction was performed after the patient’s vital signs were stable. Anesthesia was induced with sufentanil citrate injection (Yichang Humanwell Pharmaceutical Co., Ltd., H20054171, specification: 50 μg/mL) at 0.1 μg/kg through intravenous injection. After 3 minutes of anesthesia induction, the patients were anesthetized. Patients in the control group were anesthetized with ciprofol (Liaoning Haisco Pharmaceutical Co., Ltd., H20200013, specification: 50 mg/20 mL) at 0.4 mg/kg through slow intravenous injection; those in the observation group were anesthetized with remimazolam besylate (Yichang Humanwell Pharmaceutical Co., Ltd., H20200006, Specification: 25 mg) at 0.25 mg/kg through slow intravenous injection. PGE was performed when the Modified Observer’s Assessment of Alertness/Sedation score was ≤ 2. During the operation, if the patients showed body movement and obvious swallowing movements, additional anesthetic drugs (0.2–0.5 mg/kg of ciprofol or 3 mg/time of remazolam besilate) could be supplemented as needed until the patients recovered smoothly. After finishing PGE, the patients were sent to the anesthesia recovery room.

### Observation indicators

#### Clinical data collection

Clinical data, including age, gender, body mass index (BMI), and ASA grade, of all subjects were collected.

#### Anesthesia effects

Anesthesia onset time, gastrointestinal endoscopy time, recovery time, as well as anesthesia recovery room stay time of the two groups, were recorded.

#### Analgesic effects

After 30 minutes of examination, the numerical rating scale (NRS)^19^ was employed to evaluate the analgesic effects of the patients. NRS was divided into 0–10 grades, with 0–3 for mild pain, 4–6 for moderate pain, and 7–10 for severe pain. Analgesic effective rate = (mild pain + moderate pain) cases/total cases × 100%.

#### Hemodynamics

Mean arterial pressure (MAP), heart rate (HR), and oxygen saturation (SpO_2_) of the two groups were recorded before anesthesia induction (T_0_), at the time of gastroscopy reaching the throat (T_1_), 3 minutes after gastroscopy reached the throat (T_2_), and at the end of gastroscopy (T_3_).

#### Cognitive function

At 0.5 h after the patient awoke from anesthesia, mini-mental state examination (MMSE)^20^ was implemented to evaluate the cognitive function of the patients after awakening. There were 30 questions in MMSE. The correct answer to each question scored 1 point, and the wrong answer or not knowing scored 0 point, with scores ranging from 0 to 30 points. The higher the MMSE scores, the better the cognitive function.

#### Adverse reactions

Adverse reactions including nausea and vomiting, hypotension, dizziness, injection pain, bradycardia, and respiratory depression of the two groups were recorded.

#### Statistics

GraphPad Prism 6.01 software (Graph Pad Inc., La Jolla, CA, United States of America) was utilized for data analysis. Measurement data were depicted as mean ± standard deviation (*x* ± s), and t test was conducted for comparison between groups. Numeration data were depicted in the form of rate (%), and the χ^2^ test was implemented for comparison. *P* < 0.05 was considered statistically significant.

## Results

### Clinical data

No significant difference was found in age, gender, BMI, ASA grade and educational level between the two groups (*p*> 0.05) (Table 1).

**Table 1:** Clinical data between two groups.

Indicators		The control group (n = 31)	The observation group (n = 31)	χ2/t value	*p*-value
Age (years old)		69.84 ± 5.20	69.26 ± 5.38	0.432	0.667
Gender	Male	18 (58.06%)	17 (54.84%)	0.066	0.798
	Female	13 (41.94%)	14 (45.16%)		
Body mass index (kg/m^2^)		25.00 ± 3.40	24.85 ± 3.90	0.167	0.868
American Society of Anesthesiologists grade	I grade	11 (35.48%)	14 (46.16%)	0.603	0.437
	II grade	20 (64.52%)	17 (54.84%)		
Educational level	Elementary school level	7 (22.58%)	9 (29.03%)	0.37	0.831
	Middle school level	6 (19.35%)	5 (16.13%)		
	High school and above level	18 (58.06%)	17 (54.84%)		

Source: Elaborated by the author.

### Anesthesia effects

The anesthesia onset time, recovery time, as well as anesthesia recovery room stay time in the observation group, were shorter versus those in the control group (*p* < 0.05). The gastrointestinal endoscopy time and number of additional medications exhibited no difference between the two groups (*p* > 0.05). The results demonstrated that remimazolam besylate had a good anesthetic effect on PGE in the elderly (Table 2).

**Table 2:** Anesthetic effects between the two groups.

Indicators	The control group (n = 31)	The observation group (n = 31)	*t* value	*p*-value
Anesthesia onset time (s)	49.58 ± 4.98	40.65 ± 4.92	7.106	< 0.001
Gastrointestinal endoscopy time (min)	18.45 ± 3.45	18.32 ± 3.71	0.142	0.888
Recovery time (min)	9.29 ± 1.04	8.00 ± 1.07	4.829	< 0.001
Anesthesia recovery room stay time (min)	30.97 ± 4.20	23.45 ± 5.14	6.303	< 0.001
Number of additional medications	0.61 ± 0.67	0.58 ± 0.72	0.183	0.855

Source: Elaborated by the author.

### Analgesic effects

A higher effective rate of analgesia in the observation group (96.77%) was noted versus in the control group (80.65%) (*p* < 0.05). The results unraveled that remimazolam besylate had a good analgesic effect on PGE in the elderly (Table 3).

**Table 3:** Analgesic effects between the two groups.

Indicators	The control group (n = 31)	The observation group (n = 31)	χ^2^ value	*p*-value
Mild pain	10 (32.26%)	14 (45.16%)	-	-
Moderate pain	15 (48.39%)	16 (51.61٪)	-	-
Severe pain	6 (19.35%)	1 (3.23٪)	-	-
Analgesic effective rate	25 (80.65%)	30 (96.77٪)	4.026	0.045

Source: Elaborated by the author.

### Hemodynamics

MAP, HR and SpO_2_ levels in the two groups reduced first and then raised. No difference in MAP, HR and SpO_2_ levels was exhibited between the two groups at T_0_ (*p* > 0.05). At T_1_, T_2_, and T_3_, MAP and HR in the observation group were higher versus in the control group (*p* < 0.05). At T_1_ and T_2_, SpO_2_ in the observation group was higher in contrast with SpO_2_ in the control group (*p* < 0.05). The results unearthed that remimazolam besylate could improve the hemodynamics of elderly patients undergoing PGE (Table 4).

**Table 4:** Hemodynamics between the two groups.

Indicators		The control group (n = 31)	The observation group (n = 31)	*t* value	*p*-value
MAP (mmHg)	T_0_	87.52 ± 4.72	87.13 ± 4.28	0.338	0.736
	T_1_	70.23 ± 4.38	74.84 ± 3.39	4.639	< 0.001
	T_2_	74.06 ± 4.00	78.39 ± 3.20	4.698	< 0.001
	T_3_	81.48 ± 2.93	85.00 ± 3.00	4.668	< 0.001
HR (times/min)	T_0_	79.77 ± 4.75	80.23 ± 4.99	0.365	0.716
	T_1_	63.52 ± 4.46	66.39 ± 4.17	2.62	0.011
	T_2_	67.35 ± 5.10	70.77 ± 4.52	2.792	0.007
	T_3_	74.29 ± 3.46	79.61 ± 3.58	5.96	< 0.001
SpO_2_ (%)	T_0_	98.65 ± 0.84	98.61 ± 0.76	0.159	0.875
	T_1_	92.55 ± 2.77	94.97 ± 2.65	3.515	0.001
	T_2_	96.52 ± 1.34	98.35 ± 0.91	6.315	< 0.001
	T_3_	98.61 ± 0.62	98.87 ± 0.67	1.579	0.12

MAP: mean arterial pressure; HR: heart rate: SpO_2_: oxygen saturation. Source: Elaborated by the author.

### Cognitive function

MMSE scores of the observation group (28.42 ± 0.50) were higher versus those of the control group (25.19 ± 0.95) (*p* < 0.05). The results unraveled that remimazolam besylate had less effects on the cognitive function of elderly patients undergoing PGE.

### Adverse reaction

The total incidence of adverse reactions exhibited no difference between the two groups (*p* > 0.05). The results unearthed that remimazolam besylate had good safety in PGE in the elderly (Table 5).

**Table 5:** Adverse reactions between the two groups.

Indicators	The control group (n = 31)	The observation group (n = 31)	χ^2^ value	*p*-value
Nausea and vomiting	2 (6.45%)	1 (3.23%)	-	-
Hypotension	2 (6.45%)	1 (3.23%)	-	-
Dizziness	3 (9.68%)	2 (6.45٪)	-	-
Injection pain	2 (6.45%)	1 (3.23%)	-	-
Bradycardia	1 (3.23%)	1 (3.23٪)	-	-
Respiratory depression	1 (3.23%)	0 (0.00٪)	-	-
Total incidence rate	11 (35.48%)	6 (19.35٪)	2.026	0.155

Source: Elaborated by the author.

## Discussion

PGE is being increasingly and clinically utilized, and sedation management and options are important during the endoscopy procedures, which can not only reduce patient discomfort, but also facilitate high disorder detection rates^14^. This study focused on the efficacy and safety of remimazolam besylate and ciprofol in elderly patients undergoing PGE.

As previously reported, remimazolam is a safe and effective sedative drug with predictable sedative duration and rapid recovery in the process of gastrointestinal endoscopy^21^. It is demonstrated that remimazolam possesses better safety and efficacy for gastrointestinal endoscopy sedation, and remimazolam has an association with higher procedure success and lower need for rescue medication, as well as shorter total and delayed recalls^22^. Remimazolam tosilate can allow faster recovery from sedation, and patients who received remimazolam tosilate take shorter time to fully alert^12^. It is also reported that remimazolam has the ability to achieve equivalent anesthetic and analgesic impact in elderly patients undergoing hip replacement^23^.

In our paper, we compared the anesthesia and analgesic effects between the two groups, and found that the anesthesia onset time, recovery time, as well as anesthesia recovery room stay time in the observation group, were shorter versus the control group. Moreover, the effective rate of analgesia in the observation group was higher versus that in the control group. All these demonstrated that remimazolam besylate had a good anesthetic and analgesic effect on PGE in the elderly.

A previous study has revealed that the utilization of remimazolam tosilate leads to less frequent hemodynamic events^11^. In addition, remimazolam possesses lower hemodynamic effects during general anesthesia, and a lower delta HR and delta MAP display more stable hemodynamic features of remimazolam^7^. Moreover, remimazolam besylate in combination with alfentanil is demonstrated to be a safe and effective sedation option for patients undergoing painless gastroscopy, which can not only avoid deep sedation and large hemodynamic fluctuations, but also have fewer adverse reactions and reduced HR and blood pressure^24^. In our study, it was found that MAP, HR and SpO_2_ levels of the two groups reduced first and then raised. At T_1_, T_2_, and T_3_, MAP and HR in the observation group were higher; at T_1_ and T_2_, SpO_2_ in the observation group was higher, in contrast with that in the control group. The results unearthed that remimazolam besylate could improve the hemodynamics of elderly patients undergoing PGE.

The MMSE scores of the remimazolam group are higher than the propofol group^23^. Patients who received remimazolam are reported to have higher MMSE scores^25^. In our paper, it was found that the MMSE scores of the observation group were higher in contrast with the control groups. The results unraveled that remimazolam besylate had less effects on the cognitive function of elderly patients undergoing PGE. A previous study has reported that remimazolam can lead to lower risks of bradycardia, hypoxemia, and injection pain, and there are no differences of statistical significance in other adverse reactions^26^. In our paper, we unearthed that remimazolam besylate had good safety in PGE in the elderly, and the total incidence of adverse reactions presented no difference between the two groups.

However, this study has certain limitations, such as not considering whether the patients’ educational level influences MMSE scores, not conducting a longer follow-up tracking (with follow-up ending upon hospital discharge, potentially missing post-discharge adverse reactions), and having a relatively small sample size.

## Conclusion

In summary, this research demonstrated that, versus ciprofol, remimazolam besylate has better anesthetic and analgesic effects, more stable hemodynamics, less impact on patients’ cognitive function, and good safety in elderly patients undergoing PGE. Additionally, this researchlays a foundation for further exploration of the efficacy and safety of remimazolam besylate in elderly patients undergoing PGE. To enhance the robustness of these findings, a larger, multicenter, double-blind randomized controlled study is necessary in the future.

## Data Availability

The data will be available upon request.
